# Criminal punishment and violent injury in Minnesota

**DOI:** 10.1186/s40621-021-00303-3

**Published:** 2021-03-15

**Authors:** N. Jeanie Santaularia, Ryan Larson, Christopher Uggen

**Affiliations:** 1 Division of Epidemiology and Community Health, 1300 2nd Street S, Minneapolis, MN 55454 USA; 2 Minnesota Population Center, University of Minnesota, 225 19th Ave S #50th, Minneapolis, MN 55455 USA; 3 Department of Sociology, University of Minnesota, 909 Social Sciences Building, 267 19th Ave S, Minneapolis, MN 55455 USA

**Keywords:** Criminal punishment, Violent injury, Child abuse, Hospital discharge data

## Abstract

**Background:**

Violence is one of the leading causes of injury and death in the United States. One-way society attempts to eliminate violence is through criminal punishment. Yet, in many contexts, punishment fails to reduce violence and may cause other harms. Current research on violence often suffers from same-source bias which can produce spurious associations. This study assesses the associations of different forms of criminal punishment (monetary sanctions, incarceration, and probation) with violent injuries in two unique datasets.

**Methods:**

This study examines a unique combination of hospital discharge data and court administrative data, two Minnesota county-level data sources. First, we assess the spatial distribution of the three criminal punishment variables and two violent injury variables, violent injury overall and violent injury in children by county from 2010 to 2014, using Moran’s I statistic and Local Indicators of Spatial Autocorrelation. Then we assess the association of criminal punishment on violent injury and child abuse injury using a two-way fixed effects panel models.

**Results:**

Child abuse injuries are relatively rare in our data but are significantly concentrated geographically, unlike violent injuries which are more dispersed throughout Minnesota. Incarceration and probation are significantly geographically concentrated in similar regions while monetary sanctions are not geographically concentrated. We find a link between probation loads and violent injury, specifically, with a 1 day increase in per capita probation supervision associated with a 0.044 increase in violent injury incidence per 1000 people. In contrast, monetary sanctions and incarceration loads have little association with either violent injury or child abuse injury incidence.

**Conclusions:**

Criminal punishment is intended to reduce harm in society, but many argue that it may bring unintended consequences such as violence. This study finds that county-level probation has a modest positive association with county-level violent injury rates, but monetary sanctions and incarceration are less associated with violence injury rates. No measure of criminal punishment was associated with a reduction in violence. This study addresses a gap in previous literature by examining the association of punishment and violence in two unrelated datasets. High rates of criminal punishment and violent injury are both urgent public health emergencies. Further individual-level investigation is needed to assess potential links.

**Supplementary Information:**

The online version contains supplementary material available at 10.1186/s40621-021-00303-3.

## Background

Punitive social policies led to an increase in criminal punishment since the mid-1970s, which have had wide-ranging social impacts (Uggen & Manza, 2002; Lyons & Pettit, 2011; Wildeman & Wang, 2017; Wildeman et al., 2019). Criminal punishment includes but is not limited to: monetary sanctions (fines, fees, and restitution), incarceration (in prisons or jails), and community supervision (including probation and parole). Each of these forms of punishment affects different domains of health and social life, such as the effect of monetary sanctions on financial status, the effects of incarceration on freedom of movement, and the effect of probation supervision on voting eligibility and other collateral consequences of criminal records.

One potential cause and consequence of criminal punishment is violence, which is supported by theory and research. With regard to the latter, strain (Agnew et al., 2002) and deterrence (Lawrence et al., 1992) theories suggest that punishment may have direct impacts on violent injuries in adults and children. The specific mechanisms linking punishment and injury vary with the type and severity of punishment. First, even the relatively modest fines and fees that are routinely imposed for traffic and criminal offenses impose a disproportionate burden and financial strain on the poor (Schnittker & John, 2007). At the individual-level, strain can increase negative emotions such as anger, which in turn can engender violence. Strain can also operate at a community-level, with community characteristics (e.g., inequality) shaping the amount of strain engendered in the community, the selection and retention of strained individuals, and the likelihood of violent or criminal responses to strain (Agnew, 1999). Second, over 5 million Americans are currently serving sentences in the community on probation or parole supervision. Although supervision is a less restrictive alternative than incarceration, it also imposes substantial costs, consequences, and strains for probationers and their families (Phelps, 2017). Lastly, the U.S. incarcerates over 2.1 million people in prisons and jails, impacting approximately 1 in every 100 adults and imposing consequential emotional and financial strains on justice-involved populations (Sharkey, 2018; Travis et al., 2015). The research on punishment and subsequent violence often does not speak directly to community-level injury rates (Wildeman et al., 2019; Wildeman et al., 2018), though recent studies have explored associations between parental incarceration and children’s violence (Wildeman & Turney, 2014). To date, none have looked beyond incarceration to examine monetary sanctions and child maltreatment.

Violence represents a ubiquitous and serious public health problem in the United States. In 2010, there were an estimated 5.2 million violent victimization experiences among U.S. residents 12 or older (Truman, 2011). The impact of violence can be lifelong, with numerous impacts on physical and mental health (Macmillan, 2001). Despite its importance, however, serious methodological concerns surround the surveillance of U.S. violence (Ruiz-Pérez et al., 2007; Shepherd & Sivarajasingam, 2005). First, violent victimization data are collected in systems that are subject to systematic racial and class biases (Maloney et al., 2017; Putnam-Hornstein et al., 2013), and under-reporting is well documented (Ewigman et al., 1993; Schnitzer et al., 2004). For example, the primary function of Child Protection Services (CPS) and the National Child Abuse and Neglect Data System (NCANDS) is to stop violence against children. Typically, violence against children is reported to a local or state agency that follows up on the report to substantiate and act upon it. Families identified in CPS data are often highly marginalized and surveilled in society, which likely leads to over-reporting in these communities and under-reporting in more privileged communities (Putnam-Hornstein et al., 2013). Second, some violence research suffers from the same-source bias that occurs when both the exposure and outcome are drawn from a single source (e.g., National Crime Victimization Survey), which can produce spurious associations due to correlated measurement errors from the self-report. This study seeks to address these methodological concerns.

Hospital discharge data are traditionally used for billing but have also been utilized for public health surveillance of violence as well as other morbidities (Mok et al., 2010; Scott et al., 2009). One of the benefits of using hospital discharges to measure violence is that they do not depend on recall or bias to the same extent as more traditionally used self-reported data or police data. Therefore, to contribute to research on punishment and violence (Gray et al., 2017), this study assesses the spatial patterning of both violent injury and punishment, as well as association of violent injury with three types of criminal punishment: monetary sanctions, probation, and incarceration. Hospital discharge data are used to operationalize violence, which reduces biases arising from official enforcement, detection, and arrest. This study is the first to examine the association of *different types* of punishment and injuries in adults and children caused by violence. The primary hypotheses to be tested are whether high rates of punishment are associated with greater violent injury rates (by imposing strain) or, alternatively, with lower violent injury rates (by imposing the supervision associated with probation, or the incapacitation associated with incarceration).

## Methods

### Data

#### Minnesota hospital claims data

This study uses hospital administrative data obtained through the Minnesota Hospital Association (MHA) from 2010 to 2014. This database includes inpatient and outpatient data for each patient encounter with a health care provider and includes the diagnosis (International Classification of Diseases codes).

International Classification of Diseases (ICD) codes are used to describe the diagnosis of the condition being treated. ICD-9 codes are the main codes included in administrative datasets, because they are required for billing and reimbursement. E-codes are modifiers to ICD-9 codes that describe when and where the injury happened, to whom or by whom, and how. V-codes, also known as history codes, provide information about the history of the injury ((Falen & Liberman, 2005) p9). Cross-sectional MHA data on ICD-9, E-codes, and V-codes are used to measure cases of violence for this study. The spatial location of each injury was determined by the injured individual’s county of residence. Over our five-year observation period, 12.4% of the cases are missing location data and are excluded from the analysis.

#### Minnesota court administrative data

The Minnesota Court Administrator’s office (MCAO) provides data on all Minnesota criminal court events from 2010 to 2014. The spatial location of each case was determined by the county in which the case was filed, which contained no missing values. Year-specific county-level means were calculated based on aggregate, case-level, monetary sanction, confinement, and probation amounts. All punishment measures from the MCAO data are expressed per county resident.

#### Sociodemographic data

The American Community Survey (ACS) provides estimates of county-level covariates from the 2010–2014 to adjust for time-varying within-county changes that could confound the punishment-violence relationship (Sampson, 1997; Ulmer & Johnson, 2004). We use ACS 5-year “rolling window” estimates, constructed from the current year and each of the previous 4 years (e.g., 2006–2010). These 5-year estimates provide coverage across all Minnesota counties, resulting in a larger sample size as compared to the 1-year ACS estimates and more reliable indicators of variation across time. These measures include racial composition, gender, age, marriage rates, SSI/SNAP benefits, percent holding at least a bachelor’s degree of education, and the percentage of individuals out of work.

### Variable construction

#### Violence

ICD-9 codes, E-codes, and V-codes that indicate a diagnosis of violent injuries overall and child abuse injuries (a subset of violent injuries overall) are listed in Table 1. The county-year-level sums of each of these are divided by the total population in each respective count^1^. This number is multiplied by 1000 to yield an incidence rate of violent injuries and child abuse injuries.

**Table 1:** Codes Used to Define Violence

	Adult	Child	Description of Mechanism	
**Abuse**	995.8	995.5	Abandonment	E904.0,E968.4
Emotional	995.82	995.51	Hunger/starvation	E968.4
Multiple forms	995.85	995.59	Deprivation	E968.4
Neglect (nutritional)	995.84	995.52	Rape	E960.1
Physiological	995.82	995.51	Adult Abuse by perpetrator	E967.0-E967.9
Sexual	995.83	995.53	Child Abuse by perpetrator	E967.0-E967.9
Physical	995.81	995.54	Legal Intervention	E970-E977, E979.7, E997.2
Shaken Infant Syndrome		995.55	Assault/Homicide	E960.0–.1, E961,E962(.0–.9),E963, E964,E965(.0–.9), E966, E967(.0–.9), E968(.0–.5,.8,.9),E969
**Maltreatment**			Abuse by Perpetrator	V61.12,V61.22,V62.83
	995.8	995.5	Victim of Abuse	V61.12,V61.22,
Emotional	995.82	995.51	Rape	V15.41, V71.5
Multiple forms	995.85	995.59	Abuse	V71.81
Neglect (nutritional)	995.84	995.52	Neglect	V71.81
Physical	995.81	995.54	Criminal assault	V71.6
Shaken Infant Syndrome		995.55	Suspected	V71.6, V71.81
Physiological	995.82	995.51	Observation for abuse	V7181
Sexual	995.83	995.53	History of physical/emotional abuse	V15.41,V15.42
**Syndrome Battered**				
Adult/Spouse	995.81			
Baby or child		995.55		

#### Criminal justice debt

The county-year-level sums of case-level legal financial obligations are divided by the total population in the respective county-year. This provides an ‘LFO load’ measure representing the average monetary sanction ordered per capita in the county-year. These LFOs consist of fines, fees, and restitution amounts as sentenced.

#### Probation

The county-year-level sums of probation days ordered are divided by the total population in the respective county-year, yielding a per capita measure of ordered probation.

#### Incarceration

The incarceration measure is the sum of confinement days ordered divided by the total population in each county-year, providing a per capita indicator of ordered confinement. The entire ordered confinement sentence is included, regardless of any days of credit or days stayed by a judge.

### Analytic strategy

After merging MHA, MCAO and ACS data together in a county-year data set, the initial analysis assessed the spatial distribution of independent and dependent variables by county from 2010 to 2014, using Moran’s I statistic and Local Indicators of Spatial Autocorrelation (LISA). Moran’s I is a global statistic for detecting spatial clustering, testing whether the null hypothesis of a random distribution of a measure across spatial units can be rejected (Bivand et al., 2008). Moran’s I tests whether the levels of injury or punishment in Minnesota counties are correlated with the levels in a particular county’s spatial neighbors. The spatial weights matrix (i.e., the “neighbors”) was determined by first-order queen contiguity, which defines neighbors as counties that share either a common border or common vertex (i.e., a “corner”). LISA decompose Moran’s I into the contribution made by each spatial unit to the statistic, which helps identify which counties contribute significantly more than their randomly expected share to the global spatial autocorrelation (Anselin, 1995). LISA statistics, as compared to Moran’s I, are local statistics, will aid in identifying “hot” or “cold” counties in terms of their relationship to levels of injury or punishment in neighboring counties.

To examine the association of modes of punishment on violence, this analysis uses a two-way fixed effects panel model that relates changes in counties’ violence incidence rates (*Y*_*ct*_) over time to their change in punishment loads (*P*_*ct*_). We include county fixed effects (*γ*_*c*_) to capture average, time-stable, unobserved influences on each county’s outcome and year fixed effects (*γ*_*t*_) to capture average, unobserved influences common across counties within each year, such as statewide punishment policy or hospital protocol changes:

$$ {Y}_{ct}=\beta {P}_{ct}+\alpha {X}_{ct}+{\gamma}_c+{\gamma}_t+{\varepsilon}_{ct} $$

Time-varying county-level covariates are included (*X*_*ct*_) to capture within-county observed influences to further isolate the associations with the punishment indicators. Despite the autocorrelation detected at the county-level (see below), Moran’s I tests of the residuals of our fixed effect models indicate no spatial dependence after accounting for fixed effects and our time-varying covariates. Because our models are estimated on population data rather than sample data, our discussion of multivariate models focuses on estimation and patterns of results, rather than significance testing (Lane & Nelder, 1982). All analyses are conducted in the statistical package R (R: The R Project for Statistical Computing, n.d.).

## Results

**Fig. 1:**
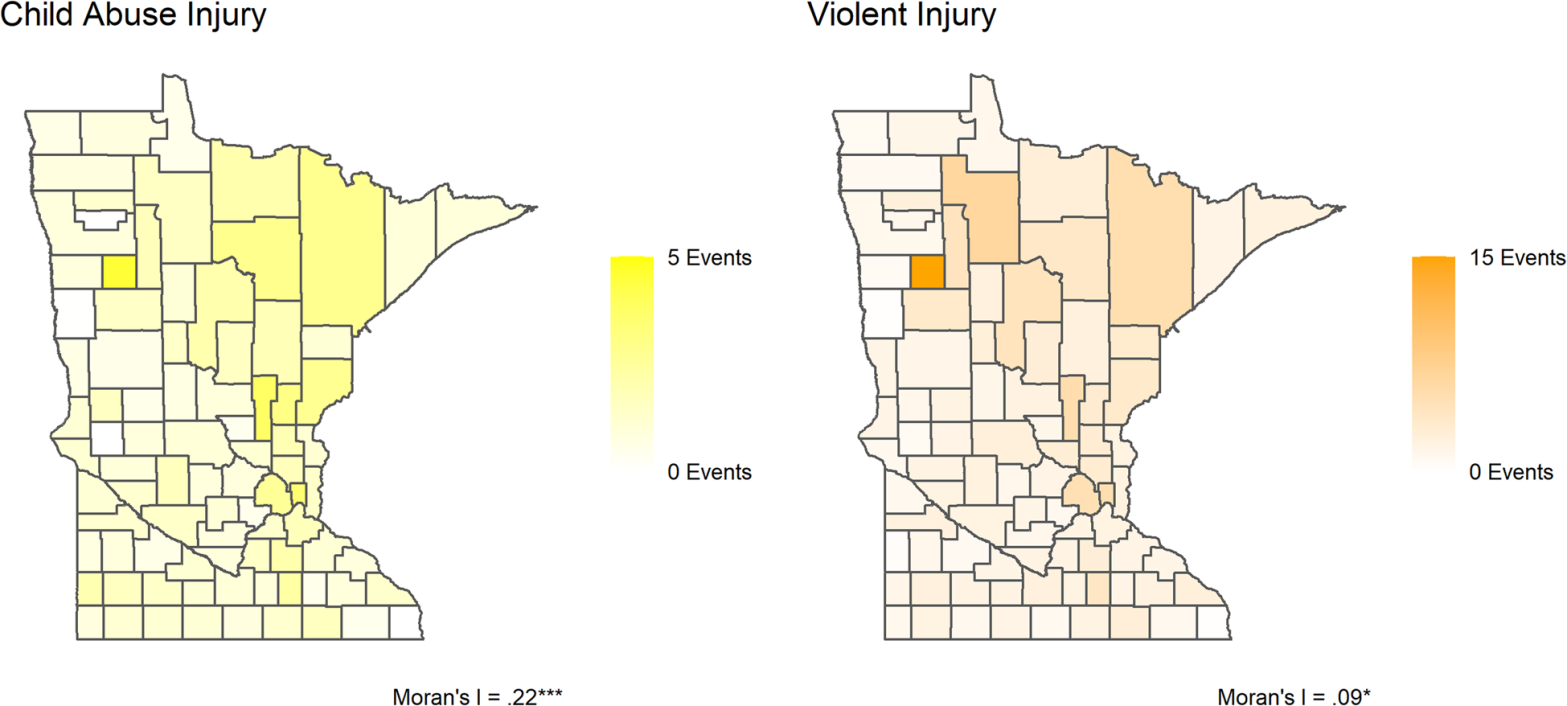
MHA Data by County, 2010–2014

Figure 1 depicts the MHA administrative data with county-level child abuse injury and violent injury incidence aggregated over the period from 2010 to 2014. Rates of violent injuries (2.31 per 1000) are higher overall than rates of child abuse injuries (1.29 per 1000). Statistically significant Moran’s I statistics for both child abuse injury and violent injury indicate the presence of spatial autocorrelation across Minnesota counties. Although child abuse injury incidence is more rare, it is more geographically concentrated than violent injury (Moran’s I = .22, *p* < .05). For child abuse, the northeastern counties (e.g., St. Louis) and urban counties (e.g., Hennepin) have slightly higher child abuse injury incidence rates as compared to the eastern and southern counties. LISA statistics (Supplementary Figure 1) indicate significant hotspots of spatial autocorrelation in the northeastern “Iron Range” area, as well as a significant coldspot in Clay county on the western border. The square, darkly-shaded Northwestern county of Mahnomen, part of the White Earth Indian Reservation, exhibits a high incidence of both child abuse injury and violent injury.

Similar patterns exist for all violent injury, albeit with lower levels of spatial autocorrelation overall (Moran’s I = .09, *p* < .05). Northeastern and urban counties have slightly higher incidence rates, with Mahnomen county again showing the highest rate overall. LISA statistics indicate three hotspot counties with significant positive autocorrelation relative to their neighbors (Itasca, Clearwater, and Becker), all located in the northern half of the state.

**Fig. 2:**
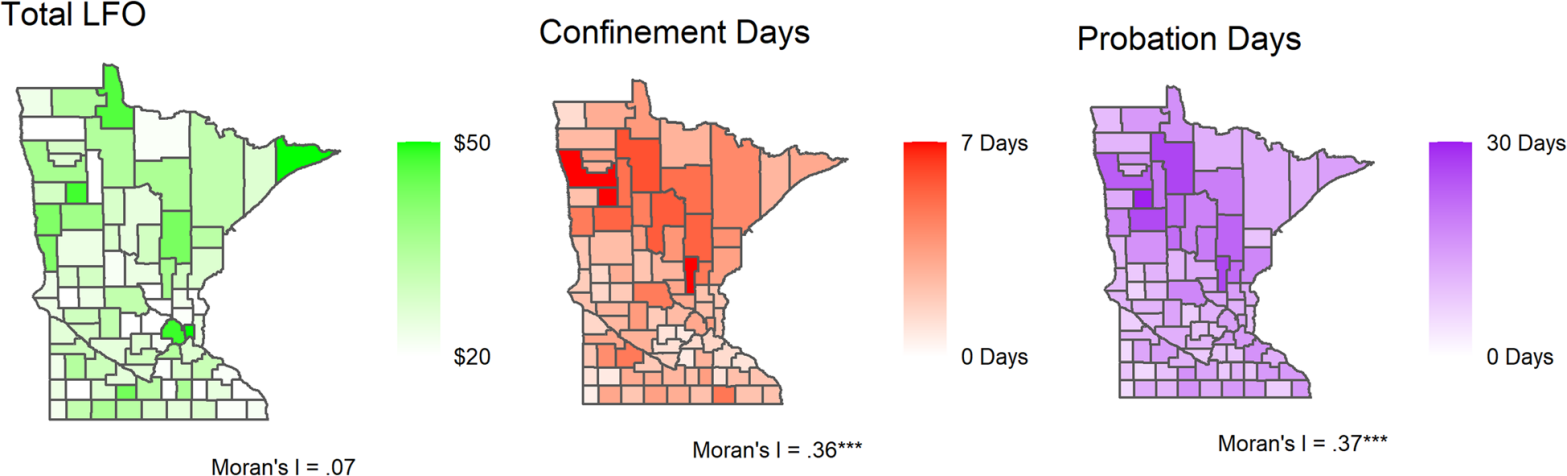
MCAO Data by County, 2010–2014

Figure 2 shows the spatial distribution of the focal punishment variables across Minnesota. In terms of total criminal justice debt, the urban counties of Hennepin and Ramsey have the highest per capita amounts, with select northern counties having higher than average loads as well. Notably, the suburban counties surrounding the Twin Cities metro area show markedly lower criminal justice debt per capita. A global test of spatial autocorrelation is statistically nonsignificant (Moran’s I = .07, *P* > .05), suggesting limited spatial autocorrelation in financial punishment. In contrast, confinement per capita has a significant level of spatial autocorrelation (Moran’s I = .36, *p* < .05). Northern counties have, on average, slightly higher confinement loads as compared to the southern parts of the state, and LISA statistics show ten significant hotspots of confinement across the northern counties, as well as significant coldspots in the southwestern suburban counties (e.g., Carver, Scott). Probation loads, although higher per capita relative to confinement (13.76 vs. 2.99), are comparably autocorrelated across counties relative to confinement loads (Moran’s I = .37, *p* < .05). LISA measures show comparable probation hotspots in the northern part of Minnesota, but do not indicate a cluster of coldspots in the southwestern metro as in confinement.

Table 2 presents fixed-effects panel models regressing violent injury incidence onto the three focal punishment indicators. Each model includes two-way county and year fixed effects, as well as all time-varying controls. In model 1, the criminal justice debt model, a one hundred dollar increase in county criminal justice debt per capita is associated with a .05 per 1000 increase of violent injury incidence. While the estimate is non-significant, it is in the anticipated positive direction as suggested by strain theories. Punitive financial practices thus show a modest positive association with the incidence of violent injuries.

**Table 2:** Panel Models of Violent Injury Incidence per 1000, 2010–2014

	(1)	(2)	(3)
LFO per Capita	0.0005 (−0.009|0.010)		
Confinement Load		0.085 (−0.002|0.172)	
Probation Load			0.044 (0.019|0.069)
Percent Hispanic	−0.201 (− 0.452|0.049)	−0.200 (− 0.447|0.048)	−0.154 (− 0.400|0.091)
Percent Black	− 0.744 (−1.026|-0.461)	−0.722 (−1.004|-0.441)	−0.710 (− 0.988|-0.432)
Percent Other	− 0.069 (− 0.233|0.096)	−0.067 (− 0.230|0.096)	−0.074 (− 0.235|0.088)
Percent Native American	−1.739 (−2.002|-1.477)	−1.737 (− 1.996|-1.478)	−1.611 (− 1.878|-1.345)
Percent Biracial	−0.596 (− 0.816|-0.375)	−0.577 (− 0.796|-0.359)	−0.538 (− 0.755|-0.320)
Percent Asian	0.003 (− 0.308|0.313)	0.005 (− 0.303|0.314)	0.059 (− 0.248|0.365)
Percent HPI	−0.803 (− 1.589|-0.016)	−0.795 (− 1.577|-0.013)	−0.824 (− 1.596|-0.052)
Marriage Rate	− 0.151 (− 0.291|-0.012)	−0.159 (− 0.298|-0.020)	−0.159 (− 0.296|-0.022)
Median Age	− 0.026 (− 0.189|0.137)	−0.035 (− 0.196|0.127)	−0.029 (− 0.188|0.130)
Percent SSI/SNAP	− 0.177 (− 0.300|-0.053)	−0.169 (− 0.292|-0.046)	−0.174 (− 0.295|-0.053)
Percent Bachelor’s Degree	−0.117 (− 0.260|0.025)	−0.110 (− 0.250|0.030)	−0.079 (− 0.219|0.061)
Percent Male	0.106 (− 0.143|0.356)	0.098 (− 0.151|0.346)	0.108 (− 0.137|0.353)
Percent No Work	0.041 (− 0.055|0.137)	0.037 (− 0.058|0.133)	0.045 (− 0.050|0.139)
County FE	Yes	Yes	Yes
Year FE	Yes	Yes	Yes
Observations	435	435	435
*R*^2^	0.379	0.385	0.400

95% Confidence Intervals in parentheses

Model 2 shows a positive association between jail and prison confinement and injury: a 1 day increase in confinement days per capita is associated with a 0.085 (95% CI: −.002–.172) increase in violent injury incidence, although this estimate only reaches conventional levels of statistical significance in specifications using a non-institutionalized population denominator^2^. There is no evidence of an incapacitation association of incarceration on violence. In contrast, Model 3 shows a statistically significant relationship between of probation loads and violent injury incidence, with a 1 day increase in per capita probation supervision associated with a 0.044 (95% CI: .019–.069) increase in violent injury incidence per 1000 people. This positive relationship, in the presence of robust two-way fixed-effects and time-varying controls, is suggestive of either a strain or detection effect of probation. As discussed below, probation is associated with the strain and stress that leads to violent injury. At the same time, probation is also associated with supervision, which could help detect violent injury.

Table 3 presents fixed-effects panel models of child abuse incidence, using identical model specifications to those presented in Table 2. In each model, the punishment estimates are small and non-significant, suggesting that child abuse injury incidence is not associated to changes in county punitiveness. This may be due to the rarity of child abuse injury as measured in the hospital data: the child abuse injury incidence measure has a substantially lower incidence rate (1.24) as compared to all violent injury (2.31), as well as a substantially lower variance (0.98 vs. 4.77). In other words, the relative infrequency of hospital reports of child abuse injury leaves very little variation to be explained by our predictors of interest, especially after accounting for all time-stable county variation and county-stable panel impacts. In simpler fixed-effect specifications without other covariates, estimates for these punishment indicators remain small and not statistically distinguishable from zero.

**Table 3:** Panel Models of Child Abuse Injury Incidence per 1000, 2010–2014

	(1)	(2)	(3)
LFO per Capita	−0.002 (−0.014|0.010)		
Confinement Load		0.018 (−0.094|0.129)	
Probation Load			−0.014 (− 0.046|0.018)
Percent Hispanic	0.004 (−0.315|0.323)	0.012 (− 0.305|0.329)	−0.004 (− 0.323|0.314)
Percent Black	− 0.045 (− 0.405|0.315)	−0.037 (− 0.398|0.323)	−0.053 (− 0.413|0.307)
Percent Other	0.086 (− 0.123|0.296)	0.083 (− 0.126|0.292)	0.085 (− 0.124|0.294)
Percent Native American	0.198 (− 0.136|0.532)	0.206 (− 0.126|0.539)	0.164 (− 0.182|0.509)
Percent Biracial	0.062 (− 0.218|0.343)	0.073 (− 0.207|0.353)	0.050 (− 0.232|0.332)
Percent Asian	−0.029 (− 0.425|0.366)	−0.025 (− 0.420|0.370)	−0.044 (− 0.441|0.353)
Percent HPI	0.024 (− 0.978|1.026)	0.033 (− 0.969|1.034)	0.037 (− 0.964|1.038)
Marriage Rate	0.125 (− 0.053|0.302)	0.122 (− 0.056|0.300)	0.126 (− 0.051|0.304)
Median Age	0.129 (− 0.078|0.337)	0.132 (− 0.075|0.339)	0.134 (− 0.072|0.341)
Percent SSI/SNAP	−0.005 (− 0.163|0.153)	−0.001 (− 0.159|0.156)	−0.004 (− 0.161|0.153)
Percent Bachelor’s Degree	0.061 (− 0.121|0.243)	0.069 (− 0.110|0.248)	0.054 (− 0.127|0.236)
Percent Male	0.025 (− 0.293|0.344)	0.027 (− 0.291|0.345)	0.028 (− 0.290|0.345)
Percent No Work	−0.095 (− 0.217|0.028)	−0.095 (− 0.217|0.027)	−0.096 (− 0.218|0.026)
County FE	Yes	Yes	Yes
Year FE	Yes	Yes	Yes
Observations	435	435	435
*R*^2^	0.028	0.028	0.030

95% Confidence Intervals in parentheses

## Discussion

This paper set out to assess the association of overall violent injury and child abuse injury and three types of criminal punishment: monetary sanctions, probation, and incarceration. In doing so, it combined two independent data sources: Minnesota Hospital Claims Data and Minnesota Court Administrative data. We found strong clustering of both injury and punishment rates, with significant “hot spots” of spatial autocorrelation for violent injury, confinement, and probation rates amongst counties in northern Minnesota. We hypothesized that high rates of monetary sanctions and incarceration would either (1) increase violent injury rates and child abuse injury by increasing strain, or (2) decrease violent injury rates and child abuse injuries by imposing supervision via probation or incapacitation via incarceration. Instead, we observe little association between monetary sanctions and violent injury. Incarceration and probation rates are associated with an increased risk of violent injury, in keeping with the first hypothesis (and in contrast to deterrence arguments), although the coefficients are generally modest in magnitude. Child abuse injuries are not significantly associated with any of the three criminal punishment variables. The relative rarity of child abuse injuries, however, should be taken into account in considering this pattern of results.

The present study finds relatively modest or null associations between criminal punishment and violence. The extant literature had yet to examine the association of incarceration (prison and/or jail time), monetary sanctions, or probation on hospital violent injury rates. In contrast to traditional violence surveillance systems whose sole purpose is to identify violence, this analysis uses an alternative measure of victimization based on hospital injury data. Future research could move to combining hospital injuries with other indicators of violence, such as homicide and self-reported child victimization^3^.

To our knowledge, this is the first study to examine monetary sanctions and violence, therefore there is little comparison literature to assess the null finding we observe. Explanations for this null effect could involve the relatively modest rate of monetary sanctions in Minnesota relative to other states (Harris et al., n.d.), as well as countervailing mechanisms and processes, such that monetary sanctions could be correlated with an unobserved factor (such as access to counseling or other services) that offsets the impact of financial strain. Although our measures of other forms of economic hardship were limited, we saw little evidence in support of this hypothesis, despite the vast literature establishing an association between economic hardship and violence and child injury (Walsh et al., 2019). There is also a wealth of evidence on the impacts of incarceration on family and child health, including a study linking county-level jail incarceration to county-level mortality (Kajeepeta et al., 2020). Other studies, however, find relatively little association between prison and later violent crimes at the individual level (Harding et al., 2019).

Our results thus show relatively modest associations between punishment and violent injury, though this study is not without limitations. First, the use of ICD codes and hospital data means that this study is missing people who experienced violent events but did not go to the hospital. For example, while extreme neglect may bring a child or an elderly person into a hospital (due to severe malnourishment where medical intervention is necessary), less extreme neglect would not be captured. Nevertheless, violent injuries may provide a more objective indicator of violence compared to traditional violence victimization sources such as Child Protective Services or Uniform Crime Reports (UCR) (Shepherd & Sivarajasingam, 2005). Second, hospital data may oversample those with health insurance in the population. That said, for more severe or urgent injuries, people often seek care even when they lack health insurance coverage. Third, MCAO data are not representative of U.S. courts. In particular, Minnesota is a relatively low-incarceration, low fine, and high-probation state that exhibits relatively high racial disparities in punishment. Despite this, these data have the advantage of comprehensiveness, as they include the vast majority of all felony, misdemeanor, and traffic cases within that state. Fourth, this is an ecological study, therefore results can only be applied to counties and cannot be linked to individuals. Fifth, because these data are repeated cross-sections, temporality cannot be assessed with confidence. Notwithstanding these limitations, this research advances knowledge on the relationship between criminal justice sanctions and violence, two parallel public health emergencies (Koop, 1992).

Despite a drop in crime since the 1990s, violence remains one of the leading causes of injury and death in the population (Centers for Disease Control and Prevention, n.d.). This paper has examined the associations of violence with criminal punishment, which is often intended to curtail the harms from violence. In many contexts, punishment fails to reduce crime and may cause other harms (Travis et al., 2015). In this context, we find relatively modest associations with fines and incarceration, but a link between probation supervision and violent injury. An important goal of criminal punishment should be to provide treatment services that help address community needs and reduce the harms of punishment itself (Travis et al., 2015). This paper has explored one of the unintended consequences of punishment, including strain in the family or community. Although our examination does not find large county-level associations, there are compelling reasons for further investigation into these associations at the individual level, including quantitative and qualitative assessments.

## Supplementary Information

**Additional file 1.**

## Data Availability

The majority of the data that support the findings of this study are available from Minnesota Hospital Association and Minnesota Court Administrator’s office but restrictions apply to the availability of these data, which were used under license for the current study, and so are not publicly available. The remaining data from the American Community Survey are available in the [NAME] repository, [PERSISTENT WEB LINK TO DATASETS].

## Abbreviations

CPS: Child Protection Services
NCANDS: National Child Abuse and Neglect Data System
MHA: Minnesota Hospital Association
ICD: International Classification of Diseases
MCAO: Minnesota Court Administrator’s office
ACS: American Community Survey
LFO: Legal financial obligations
LISA: Local Indicators of Spatial Autocorrelation
UCR: Uniform Crime Reporting

## Footnotes

1 Rates for the reported models are based on an inclusive total population denominator, since some violent injuries occurring in institutions (e.g., jails) are treated in hospitals. As explained below, we also conducted a sensitivity analysis using the non-institutionalized population to compute denominators.

2 As a sensitivity analysis, we re-estimated these models on the non-institutionalized population (subtracting the county-year level ACS 5-Year Group Quarters population estimates from the overall population). The results parallel those reported in Table 2, except the confinement estimate in model 2 grows stronger in magnitude (.122 [95% CI: .028–.217] vs .085 [95% CI: −.002–.172]). We caution, however, that under this specification, changes in confinement may be driving changes in the numerators (violent injuries), that would not be reflected in the denominator (e.g., rising jail populations).

3 As a check on the robustness of these findings, we also examined two other indicators of violent injury: the homicide rate as reported by the Uniform Crime Reports and the proportion of students reporting that a parent or other adult has ever physically hurt them, as measured by the Minnesota Student Survey. Confinement rates show a modest positive association with both homicide and self-reported victimization, probation rates are positively associated with victimization, but not homicide, and monetary sanctions are not associated with either outcome.A one unit increase in confinement and probation loads is associated with a .45 and .20 percentage point increase in students reporting that a parent or other adult has ever hit, beat, kicked or physically hurt them in any way. In terms of homicide, confinement (lagged by 1 year to establish temporal order) is associated with a .0016 increase in homicide rates, whereas lagged probation is associated with a .0016 decline in homicide rates. Although a full analysis would be beyond the scope of this paper, these results generally corroborate our analysis of hospital injury data in terms of less extreme injuries that go undetected, but may not universally generalize to fatal forms of violence. Full models available upon request to the authors.
