# Supplementary material for: Criminal punishment and violent injury in Minnesota
Source: Inj Epidemiol. 2021 Mar 15;8:11. doi: 10.1186/s40621-021-00303-3 (PMC7958430; doi:10.1186/s40621-021-00303-3)
Supplement: Supplementary file 1 — Additional file 1. [file 40621_2021_303_MOESM1_ESM.docx]

| **Supplementary Table 1: Descriptive Statistics** | | | | | | | | |
| --- | --- | --- | --- | --- | --- | --- | --- | --- |
| Statistic | N | Mean | St. Dev. | Min | Pctl(25) | Median | Pctl(75) | Max |
|  | | | | | | | | |
| Violent Injury Incidence | 435 | 2.308 | 2.183 | 0.000 | 1.348 | 1.859 | 2.622 | 24.516 |
| Child Abuse Injury Incidence | 435 | 1.293 | 0.990 | 0.000 | 0.641 | 1.081 | 1.746 | 6.490 |
| LFO per Capita | 435 | 28.428 | 10.242 | 11.105 | 21.093 | 26.429 | 33.429 | 85.645 |
| Confinement Load | 435 | 2.991 | 1.861 | 0.489 | 1.843 | 2.627 | 3.676 | 15.751 |
| Probation Load | 435 | 13.761 | 6.374 | 3.476 | 9.767 | 12.752 | 16.118 | 57.101 |
| Percent Hispanic | 435 | 3.617 | 3.549 | 0.000 | 1.491 | 2.644 | 4.483 | 24.489 |
| Percent Black | 435 | 1.315 | 1.863 | 0.000 | 0.370 | 0.647 | 1.527 | 11.928 |
| Percent Other | 435 | 0.920 | 0.946 | 0.000 | 0.258 | 0.639 | 1.332 | 6.422 |
| Percent Native American | 435 | 1.907 | 4.887 | 0.000 | 0.283 | 0.500 | 1.225 | 41.086 |
| Percent Biracial | 435 | 1.673 | 1.014 | 0.221 | 1.042 | 1.510 | 2.040 | 8.073 |
| Percent Asian | 435 | 1.257 | 1.781 | 0.000 | 0.388 | 0.572 | 1.185 | 12.770 |
| Percent HPI | 435 | 0.046 | 0.110 | 0 | 0 | 0 | 0.04 | 1 |
| Marriage Rate | 435 | 22.235 | 2.044 | 16.278 | 21.023 | 22.498 | 23.516 | 27.595 |
| Median Age | 435 | 41.185 | 4.736 | 29.700 | 37.450 | 41.900 | 44.400 | 52.600 |
| Percent SSI/SNAP | 435 | 3.947 | 1.708 | 0.193 | 2.929 | 3.672 | 4.560 | 15.075 |
| Percent Bachelor's Degree | 435 | 10.275 | 2.938 | 5.858 | 8.516 | 9.810 | 11.130 | 20.647 |
| Percent Male | 435 | 50.121 | 0.853 | 47.247 | 49.583 | 50.069 | 50.566 | 53.563 |
| Percent No Work | 435 | 22.680 | 4.085 | 13.172 | 19.882 | 22.609 | 24.995 | 36.989 |
|  | | | | | | | | |

**Supplementary Figure 1
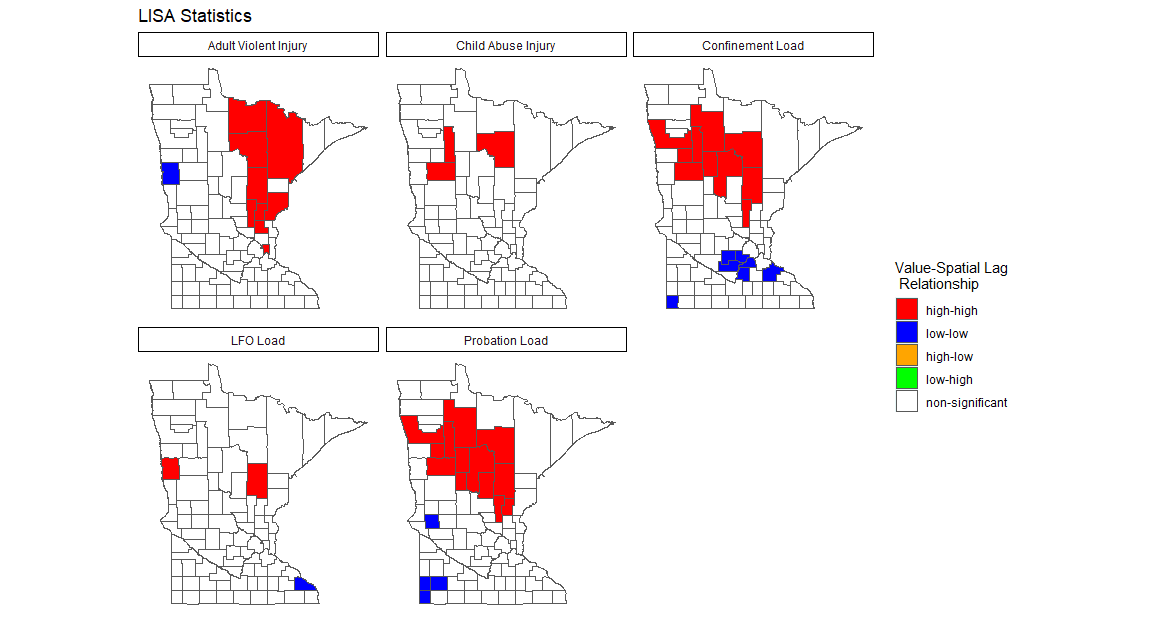
**
